# Let Us Divide Twice: A New Design of Duplex Quantitative Lateral Flow Immunoassay

**DOI:** 10.3390/s26217012

**Published:** 2026-11-05

**Authors:** Nadezhda S. Komova, Kseniya V. Serebrennikova, Anatoly V. Zherdev, Boris B. Dzantiev

**Affiliations:** A.N. Bach Institute of Biochemistry, Research Center of Biotechnology of the Russian Academy of Sciences, Leninsky Prospect 33, 119071 Moscow, Russia; nad4883@yandex.ru (N.S.K.); ksenijasereb@mail.ru (K.V.S.); zherdev@inbi.ras.ru (A.V.Z.)

**Keywords:** lateral flow immunoassay, multiplex immunoassay, cortisol, testosterone, saliva

## Abstract

**Highlights:**

**What are the main findings?**

- A duplex LFIA was developed for the simultaneous quantitative determination of cortisol and testosterone in saliva within the working ranges of 0.38–5.41 ng/mL and 27–845 pg/mL, respectively.
- The interception zone removed the cortisol recognition probe before it reached the testosterone assay zone, thereby minimizing analytical interference.

**What are the implications of the main findings?**

- The spatial organization of immunochemical reactions provides an alternative strategy for multiplexing LFIA without the use of spectrally distinct labels or complex assay designs.
- The proposed design allows for the independent optimization of biomarker assays on a single test strip that are characterized by significantly different ranges of detectable concentrations.

**Abstract:**

Simultaneous determination of salivary cortisol and testosterone levels could provide complementary information on the physiological status of the human body. However multiplex immunoassays are challenging because the two analytes occur at different concentration levels and the corresponding immunochemical reagents can interfere with one another. Here, we developed a duplex lateral flow immunoassay (LFIA) for simultaneous colorimetric determination of cortisol and testosterone. The assay was constructed on a single nitrocellulose membrane containing two spatially separated competitive immunoreaction zones. A distinctive feature of the immunoassay design was a selective interception zone that was located between the two test lines and functioned as an immunochemical sink. The optimized assay provided working ranges of 0.38–5.41 ng/mL for cortisol and 27–845 pg/mL for testosterone, with calculated detection limits of 0.18 ng/mL and 10.1 pg/mL, respectively. The analysis took 20 min and enabled the detection of both signals using a single test strip and colorimetric signal processing. Analysis of spiked artificial saliva samples yielded recoveries of 96.4–105.7%. The results demonstrate that spatial organization of immunochemical reactions may represent an alternative strategy for multiplexing LFIAs in cases where spectrally distinct labels are undesirable or when the analytical requirements of individual assays differ significantly.

## 1. Introduction

Saliva has attracted increasing interest as a biological matrix for noninvasive measurement of steroid hormones [1,2]. Compared with venous blood collection, saliva sampling is simple, minimally invasive, suitable for repeated measurements, and can be performed outside conventional laboratory settings [3,4]. It has been established that saliva contains a wide range of analytes, including hormones, cytokines, enzymes, metabolites, and immunological markers, which confirms the potential of this biological material for identifying many systemic and local diseases, as well as assessing the functional state of a person [4,5]. Measuring the concentration of steroid hormones in saliva primarily reflects their free, circulating fractions as these hormones can diffuse from the bloodstream into saliva. Although salivary steroid concentrations are substantially lower than those in serum, the magnitude of this difference varies between hormones. According to Wood [6], the salivary-to-serum concentration ratios are approximately 1:20 for cortisol (5%) and 1:90 for testosterone (1.1%). Nevertheless, saliva can provide additional information beyond that obtained from measuring total hormone levels in blood serum, which includes both free and protein-bound fractions [6,7]. These properties have made salivary hormone analysis relevant to endocrinology, stress research, chronobiology, sports science, and longitudinal monitoring. Despite these advantages, quantitative analysis of salivary biomarkers remains analytically demanding. Salivary concentrations are substantially lower than those encountered in serum, and measurements can be influenced by sampling time, salivary flow, collection devices, food intake, contamination, and other preanalytical factors. Therefore, analytical methods designed to measure biomarkers in saliva must combine sufficient sensitivity with appropriate selectivity and reproducibility.

Liquid chromatography–tandem mass spectrometry (LC-MS/MS) is a widely used method for determining hormonal profiles and metabolites in saliva with the ability to simultaneously determine multiple biomarkers of interest [1]. For example, the simultaneous determination of hormones by LC-MS/MS has been demonstrated in studies of sex hormones [8,9,10] and biochemical studies of circadian rhythms [11,12]. Nevertheless, LC-MS/MS requires expensive instrumentation, laboratory infrastructure, sample preparation, and trained personnel, which limits its application for decentralized testing.

Less labor-intensive methods of saliva diagnostics include enzyme-linked immunosorbent assays (ELISAs) [13,14,15] and chemiluminescent immunoassays (CLIAs) [16,17,18]. Immunoassays allow for the detection of a wide range of biomarkers with high accuracy at a relatively low cost, making them suitable for routine laboratory practice. Furthermore, electrochemical biosensors are becoming increasingly widespread, enabling the detection of biomarkers with high sensitivity. Low limits of detection (down to picomolar and femtomolar concentrations), rapid response, and the potential for miniaturization make electrochemical biosensors particularly attractive for identifying biomarkers in complex biological samples [19,20,21]. On the other hand, point-of-care testing offers alternative methods for detecting salivary biomarkers, aiming to reduce the burden on modern healthcare systems and provide rapid, noninvasive health monitoring and disease diagnosis [3,22,23]. In particular, the lateral flow immunoassay (LFIA) is a membrane-based immunochromatographic test strip that uses a label with unique optical properties to obtain qualitative or quantitative information about the content of a target analyte [24,25,26]. The benefits of LFIA, including rapid analysis, low cost, lack of need for expensive equipment, and the ability to integrate with digital reading systems (smartphones or portable detectors), have fueled researchers’ interest in developing membrane-based tests for noninvasive saliva diagnostics. Recent studies are aimed at integrating LFIA with various approaches to improve the performance of saliva analysis through the use of new labels [27,28] and enhancement techniques [29,30,31], magnetic preconcentration [32], the use of highly sensitive methods for detecting the signal generated by the labels [33,34] and the mobility of studies through the use of smartphone data processing [35,36].

Among the variety of LFIA tests, multiplex test systems capable of simultaneously detecting several biomarkers are of particular interest for diagnostics and monitoring human health [37,38]. However, developing multiplex LFIAs is challenging, as the concentrations of most biomarkers in saliva are low and reference intervals for complementary biomarkers often differ by several orders of magnitude [3,24,39]. Therefore, the search for new architectural solutions that retain all the advantages of point-of-care membrane tests and ensure the necessary sensitivity, as well as the absence of potential cross-reactions, is a pressing area of research. For example, Kim et al. [30] proposed a design for a dual-channel self-amplifying LFIA for the simultaneous detection of cortisol and melatonin, in which two test strips are connected by a sample pad. He et al. [40] developed a disc-like LFIA, which consisted of three independent test strips also combined into a single sample pad, for the simultaneous detection of three periodontitis biomarkers (MMP-8, IL-1β, and TNF-α) in gingival fluid.

In this study, we developed a dual LFIA for the simultaneous determination of cortisol and testosterone, selected as complementary markers of hypothalamic–pituitary–adrenal and hypothalamic–pituitary–gonadal axis activity. Measuring these parameters together is particularly relevant, as the relationship between the concentrations of these hormones can provide information about endocrine status that cannot be reliably obtained using either biomarker alone. Therefore, cortisol and testosterone analysis, including their relative balance, has been studied in sports medicine and endocrinology in the context of stress, fatigue, recovery, overtraining, and adaptive responses [41,42,43]. This determines the clinical significance of the cortisol and testosterone pair for multiplex analysis.

However, the simultaneous determination of cortisol and testosterone using LFIA presents a challenge, as the two biomarkers have distinct clinically significant concentration ranges that must be measured on a single test strip. In particular, the assessment of physiological stress, endocrine function, and anabolic–catabolic balance requires the measurement of salivary cortisol and testosterone at different concentration levels [1,44]. Typical morning salivary cortisol concentrations are approximately 4 ng/mL, decreasing to approximately 1 ng/mL in the evening, whereas salivary testosterone concentrations in men typically range from approximately 50 to 150 pg/mL [44]. However, reference intervals for both hormones may vary depending on factors such as sex, age, sampling time, physiological status, and the analytical method used.

This study proposes an LFIA that enables the determination of cortisol and testosterone through the controlled monitoring of competitive immunoreactions on a common nitrocellulose membrane. To address the challenge of detecting two target analytes across different concentration ranges, a novel test strip design featuring an interception zone positioned between the two test lines is proposed. This configuration ensures the spatial separation of the two competing immune reactions and the selective removal of the anti-cortisol monoclonal antibody–gold nanoparticle complex (CORT recognition probe) within the interception zone, which contains anti-species antibodies, before the complex reaches the testosterone detection zone. The developed LFIA platform offers two important advantages: eliminating the need for spectrally distinct labels or complex multiplex detection equipment due to the spatial separation of the immunoreactions and enabling independent evaluation of the analytical performance of two immunoassay systems despite their simultaneous operation. Thus, two biomarkers are detected using the same colorimetric principle based on gold nanoparticles as a label and quantitatively processing data from a single strip.

## 2. Materials and Methods

### 2.1. Reagents

The reagents applied in this study were the following: hydrocortisone 3-(O-carboxymethyl)oxime, N-hydroxysuccinimide (NHS), N-(3-dimethylaminopropyl)-N’-ethyl-carbodiimide (EDC), testosterone, progesterone, gold (III) chloride hydrate (HAuCl4 × 6H2O), sodium citrate, bovine serum albumin (BSA), ovalbumin (OVA), Tris, HEPES, Triton X-100, Tween-20, and artificial saliva (Sigma Chemical Co.,St. Louis, MO, USA); β-Estradiol (Shanghai Macklin Biochemical Technology Co., Ltd., Shanghai, China); cortisone and dehydroepiandrosterone sulfate (DHEA-S) (local pharmacy, Moscow, Russia); “Cortisol Saliva ELISA” kit for the determination of free cortisol in saliva and “Testosterone Saliva ELISA” kit (LDN Immunoassay and Services, Nordhorn, Germany); and other chemicals (Khimmed, Moscow, Russia). The chemicals and organic solvents applied in the experiments were of analytical grade and therefore did not require additional purification.

The antibodies used in this study were the following: the monoclonal antibodies (mAbs) to testosterone (clone XE-006), testosterone–BSA, and cortisol (clone LC-002) (Fapon Biotech, Dongguan, China).

For the fabrication of lateral flow tests, nitrocellulose membranes (type CNPH) and the GFB-R4 sample pad (Advanced Microdevices, MDI, Ambala Cantt, India), as well as CFSP223000 absorbent pad (Millipore, Bedford, MA, USA) were applied.

### 2.2. Synthesis of AuNPs and Preparation of Recognition Probes

#### 2.2.1. Synthesis of AuNPs

Gold nanoparticles (AuNPs) with a diameter of 30 nm were synthesized by reducing chloroauric acid (HAuCl_4_) with sodium citrate, as previously described [45]. Briefly, 1.5 mL of 1% (*w*/*v*) sodium citrate was added to 100 mL of a boiling 0.01% (*w*/*v*) HAuCl_4_ solution. The reaction mixture was maintained at 100 °C for 15 min and subsequently cooled to room temperature. The resulting AuNPs were stored at 4–6 °C. Before use, the AuNPs were washed by centrifugation at 7500× *g* for 15 min and resuspended in the original volume of deionized water.

#### 2.2.2. Antibody Modification and Purification

To activate monoclonal antibodies against cortisol (clone LC-002) and testosterone (clone XE-006), 10 µL of OPSS-PEG-NHS solution (2 mg/mL) was added to 500 µL of mAb-CORT or mAb-TEST (concentration of antibodies was 100 µg/mL) prepared in 100 mM carbonate buffer (pH 9.2). The mixtures were incubated for 45 min at room temperature. The modified antibodies were subsequently purified using Amicon 30K centrifugal filter units by centrifugation at 10,000× *g* and 4 °C for 10 min, with the procedure repeated 3 times using the same carbonate buffer.

#### 2.2.3. Conjugation of Antibodies with AuNPs

The mAb-AuNPs (recognition probes) were prepared according to the previously described procedure [46], with minor modifications. The purified, modified mAbs were then added to 1 mL of AuNPs to obtain a final Ab concentration of 10 µg/mL and incubated for 1 h at room temperature. Subsequently, 20 µL of an aqueous mPEG-SH solution (5 mg/mL) was added, and the mixtures were incubated for an additional 30 min. The obtained recognition probes were then collected by centrifugation at 10,000× *g* and 4 °C for 15 min. The resulting pellet was resuspended in 20 mM Tris buffer containing 0.1% BSA and 0.1% PEG-SH and stored at 4 °C until further use.

### 2.3. Characterization of AuNPs

The synthesized nanoparticles and reporters were characterized by absorption spectroscopy, dynamic light scattering (DLS), and transmission electron microscopy (TEM). Absorption spectra were recorded using a UV-2450 spectrophotometer of Shimadzu (Kyoto, Japan).

For TEM analysis, a colloidal solution of AuNPs was deposited onto 300-mesh grids coated with a polyvinyl formalin support film dissolved in chloroform. Microphotographs were acquired using a JEM-100C transmission electron microscope of JEOL Ltd. (Tokyo, Japan). The obtained images were analyzed using the Image Tool software (version 3.6, University of Texas Health Science Center, San Antonio, TX, USA).

The hydrodynamic size of AuNPs and their conjugates was determined by DLS using a Zetasizer Nano ZSP from Malvern (Worcestershire, UK) equipped with a 10 mW laser (633 nm wavelength) at 25 °C. For measurements, 100 µL of the AuNPs or recognition probes and 900 µL of deionized water were added to a quartz cuvette. Each measurement consisted of 10 scan series, and sample analysis was performed in triplicate. Statistical analysis of the DLS data was performed using Malvern software version 7.11 (Malvern, UK).

### 2.4. Synthesis of Cortisol–Ovalbumin Conjugate

The cortisol–OVA conjugate was synthesized using the carbodiimide method with the cortisol derivative, hydrocortisone 3-(O-carboxymethyl)oxime, according to the procedure described in [47]. Briefly, 200 μL of hydrocortisone 3-(O-carboxymethyl)oxime (10.3 mM) in dimethylformamide was sequentially added with 2.56 mg of N-hydroxysulfosuccinimide and 1.42 mg of 1-ethyl-3-(3′-dimethylaminopropyl)carbodiimide, and the reaction mixture was incubated for 24 h at 4 °C. After removing the precipitate from the activated cortisol derivative by centrifugation at 5000× *g*, 800 μL of OVA solution (20 mg) was added dropwise, and the mixture was incubated for 24 h at 4 °C. The resulting cortisol–OVA was dialyzed against 50 mM phosphate-buffered saline containing 100 mM NaCl, pH 7.4, and stored at −20 °C.

### 2.5. Fabrication of Duplex Lateral Flow Immunotests

All reagents were applied to the test strip using an Iso-Flow dispenser from Imagene Technology (Hanover, NH, USA). Cortisol–OVA and testosterone–BSA conjugates were immobilized as separate test lines at distances of 40 mm and 50 mm from the bottom edge of the test strip (where the sample pad was located). Cortisol–OVA and testosterone–BSA concentrations ranged from 0.25 to 1 mg/mL and 0.15 to 1 mg/mL, respectively. A selective interception zone was located between the two test lines, 4 mm above the test line containing cortisol–OVA. The interception zone contained immobilized goat anti-mouse antibodies, the concentration of which varied from 0.25 to 1 mg/mL. Protein G (concentrations varied from 0.05 to 0.2 mg/mL) was immobilized 5 mm above test line 2 containing testosterone–BSA. All loading solutions were prepared in 50 mM PBS, pH 7.4. After all reagents were applied to the nitrocellulose membrane, it was dried for 2 h at 37 °C. Finally, the sample pad and absorbent pad were fixed on the plastic support and cut into 4 mm wide strips using an Index Cutter-1 guillotine from A-Point Technologies (USA). The assembled test strips were packed into zip-lock bags containing silica gel as a desiccant and stored at room temperature.

### 2.6. Performance of the Duplex Lateral Flow Immunoassay

In the wells of a microplate, 50 μL of the sample and 1 μL of each recognition probe (OD525 = 4 for mAb (CORT)-AuNPs and OD525 = 5 for mAb (TEST)-AuNPs) were mixed and incubated for 3 min. Test strips were immersed in the solutions, incubated for 15 min, and scanned using a CanoScanLiDE 90 scanner (Canon, Tokyo, Japan) at a resolution of 600 dpi. TotalLab software (version 2009, Newcastle upon Tyne, Gosforth, UK) was applied to process the resulting images and evaluate the color intensity of colored lines.

### 2.7. Data Processing

The dependence of the color intensity (colorimetric signal) in test line 1 (TL1) or the ratio of the intensities of test line 2 to control line 2 (TL2/CL2) on the cortisol or testosterone concentration in the sample, correspondingly, was fitted using Origin software version 9.0 of OriginLab (Northampton, MA, USA) with a four-parameter sigmoidal function. The limit of detection was calculated as the concentration corresponding to the mean background signal (in the absence of analytes) minus three times its standard deviation. To obtain the aforementioned values and use them to calculate the detection limits, the blank sample was tested 10 times.

## 3. Results

### 3.1. Characteristics of AuNPs and Recognition Probes

In this study, gold nanoparticles were selected as the label to provide a colorimetric signal in duplex LFIA. Due to their ease of synthesis and stability, AuNPs are a widely used plasmonic material in the development of biosensors based on various types of transductions and signal readouts [48, 26]. Among the variety of gold nanostructures applied as colorimetric labels in LFIA, spherical AuNPs with an average diameter of approximately 30 nm were selected. This nanoparticle size is commonly used in LFIAs because it offers a practical compromise between optical signal intensity, colloidal stability, and efficient antibody conjugation. However, the optimal particle size depends on the nanoparticle composition, surface chemistry, conjugation strategy, and assay design [49,50,51,52]. Furthermore, in the development of a duplex LFIA, it is particularly important that the gold nanoparticle-based recognition probes migrate efficiently along the nitrocellulose membrane without encountering steric hindrance while maintaining sufficient signal intensity in all analytical zones.

AuNPs were synthesized by citrate reduction of chloroauric acid and subsequently functionalized with specific antibodies to obtain recognition probes. The optical and morphological characteristics of the synthesized AuNPs were evaluated by absorbance spectroscopy, TEM and DLS. The AuNPs exhibited the characteristic localized surface plasmon resonance band at 525 nm (Figure 1a), indicating the formation of nanoparticles. The TEM image showed predominantly spherical nanoparticles with relatively uniform morphology and an average diameter of 30.4 ± 3.1 nm (Figure 1b). The DLS analysis showed a hydrodynamic particle size distribution of 35 ± 4 nm (Figure 1c), indicating that the colloidal solution was free of aggregation and that the nanoparticles were suitable for subsequent functionalization with antibodies.

Following the preparation of the recognition probes, their optical characteristics showed a slight bathochromic shift (red shift) of the absorption band to 526 for CORT and 529 nm for TEST, indicating successful functionalization of the AuNPs without aggregation (see Figure 2a,b). DLS measurements revealed an increase in hydrodynamic diameter to 78.8 ± 4.3 nm for the CORT recognition probe and 72.9 ± 5.6 nm for the TEST recognition probe (Figure 2c,d). The observed increase in hydrodynamic diameter is attributed to the formation of a hydrate layer on the nanoparticle surface and confirms the successful preparation of the probes.

### 3.2. Principle of the Duplex Lateral Flow Immunoassay

The principle of a traditional competitive LFIA using a single flow on a single test strip for the simultaneous detection of target hormones is illustrated in Figure 3a. The test strip design utilizes a single nitrocellulose membrane featuring two spatially separated competitive reaction zones for the detection of cortisol (TL1) and testosterone (TL2). In this competitive assay, TL1 and TL2 contain analyte–protein conjugates. This study utilized a cortisol–OVA conjugate, prepared according to the procedure described in Section 2.4, as well as a commercially available testosterone–BSA conjugate. In the absence of cortisol and testosterone, the recognition probes migrate along the test strip and bind to the corresponding capture conjugates, resulting in the formation of two red test lines. In the presence of the target hormones, the recognition probes bind to the analytes in the solution, leading to reduced binding to the immobilized CORT-OVA and TEST-BSA conjugates and a decrease in the color intensity of the test lines.

However, the significant difference in the clinically relevant concentration ranges of the hormones being tested necessitates improvements to traditional multiplex assays. In this study, we propose a novel duplex LFIA design that integrates two analytical systems with distinct operational requirements while preventing cross-interference between the molecular probes used for analyte recognition (Figure 3b). A distinctive feature of this design is the presence of an “interception zone.” In this context, an interception zone refers to the area of the test strip where anti-species antibodies are immobilized. These antibodies bind any residual CORT recognition probe intended for the recognition of antigen (cortisol), preventing them from reaching the downstream detection zone for testosterone. In other words, they “intercept” a component that could otherwise interfere with the immunochemical reaction for testosterone. Simultaneously, the TEST recognition probe passes unimpeded through the interception zone to participate in the testosterone detection reaction. In addition to the interception zone, a distinctive feature of the duplex LFIA is the inclusion of a control line for the testosterone immunochemical reaction zone (CL2), which contains immobilized Protein G (Figure 3b). In this setup, CL2 reflects the amount of the TEST recognition probe that actually passed through the analytical system but did not bind at TL2. As the testosterone concentration increases, the amount of the TEST recognition probe available for binding in zone T2 decreases, while the corresponding fraction of unbound probe migrates to zone C2. Consequently, these two signals provide complementary information regarding the distribution of the TEST recognition probe between the analytical and control zones. Thus, the proposed design ensures the spatial separation of the two immunochemical reactions, enabling the independent determination of both analytes.

### 3.3. Evaluation of the Interception Zone

The suitability of the test strip design, incorporating an additional interception zone between the cortisol and testosterone immunochemical reaction zones, was evaluated by assessing the migration of the CORT recognition probe on test strips both with and without this zone. In the absence of the interception zone, the residual CORT recognition probe migrated to the region where TEST-BSA was immobilized. Non-specific binding of the CORT recognition probe was observed at TL2, indicating potential interference with testosterone detection (Figure 4a, without an interception zone). Introducing an interception zone between TL1 and TL2 resulted in the binding of the CORT recognition probe to immobilized anti-species antibodies, preventing its further migration to TL2 (Figure 4a, with an interception zone). It is important to note that the interception zone demonstrated selectivity for the CORT recognition probe, whereas the TEST recognition probe did not bind to the anti-species antibodies but interacted specifically with the TEST-BSA conjugate immobilized on TL2 (Figure 4b). When both recognition probes and analytes were present simultaneously, the interception zone ensured spatial separation of the components, confirming the feasibility of conducting two competitive immunochemical reactions on a single membrane (Figure 4c, with an interception zone).

The performance of the proposed LFIA design was also evaluated by analyzing standard solutions of cortisol and testosterone using test strips both with and without the interception zone (Figure 5). Figure 5 and Table 1 present calibration curves illustrating the dependence of the TL1 or TL2 signal intensity on the concentration of cortisol or testosterone, as well as the calculated analytical characteristics, respectively. As shown in Figure 5 and Table 1, the limit of detection for testosterone was higher in the absence of the interception zone compared to the test system incorporating it. The observed effect is explained by the fact that the interception zone completely removes the CORT recognition probe that did not bind during the cortisol immunoreaction, thereby eliminating any non-specific binding of this probe within the testosterone immunoreaction zone. Specifically, the limit of detection for testosterone was 0.4 ng/mL for the system without the interception zone versus 0.06 ng/mL with it. Furthermore, the calculated cross-interference (CI) in the high cortisol and low testosterone conditions was significantly reduced (from 13.8% to 3.4%).

The transition to the new test strip architecture shifted the working range for cortisol from 0.03–0.6 ng/mL to 0.3–3.9 ng/mL (Figure 5, Table 1), aligning with reference values for this biomarker in saliva. It is important to note that this shift did not lead to an increase in the coefficient of variation (CV) or the CI value. This indicates that IZ alters the working range of the cortisol assay without impairing measurement precision or introducing additional cross-interference. Moreover, the exclusion of the probe that recognizes cortisol reduces interference and allows the immunochemical system for testosterone detection to function independently.

In subsequent studies, the LFIA architecture was supplemented with a control line where immobilized Protein G interacted with the unbound TEST recognition probe. Thus, the test strip design comprised the following components, arranged from bottom to top: test line 1 (CORT-OVA), interception zone (GAMI), test line 2 (TEST-BSA), and control line 2 (Protein G).

### 3.4. Optimization of the Duplex LFIA

Assay conditions were optimized by monitoring the colorimetric responses of both test systems while varying the composition of the saliva dilution buffer, the optical density of the recognition probes, the antibody concentration in the recognition probes, and the concentration of immunoreagents immobilized on the nitrocellulose membrane. Since two competitive immunoassays were performed simultaneously on the same membrane, optimization required taking both colorimetric responses into account. The selection criterion was the achievement of the maximum signal ratio (in the absence versus the presence of the analyte) for both target substances.

Figure 6 demonstrates the dependence of the colorimetric responses on the composition of the buffers used for saliva dilution. As shown in Figure 6, the buffer composition had the most pronounced effect on the test system for testosterone determination. Therefore, to enable the simultaneous determination of both analytes, PBS was selected as the optimal buffer, as it was suitable for both systems.

Evaluation of the effect of recognition probe concentration on the colorimetric response showed that increasing the optical density of the recognition probes led to an increase in the signal in the absence of the analyte (Figure 7). The maximum signal amplitude was observed when using CORT and TEST recognition probes with optical densities of 5 and 4, respectively. An analysis of signal dynamics using recognition probes with varying degrees of antibody loading demonstrated that the optimal antibody concentration for AuNP functionalization is 10 µg/mL for both systems (Figure 8).

Next, the effect of the concentration of immunoreagents immobilized on the nitrocellulose membrane on the colorimetric response was investigated in the presence (1 ng/mL for cortisol and 200 pg/mL for testosterone) and absence of analytes. As shown in Figure 9a,b, the use of higher concentrations of immobilized conjugates (1 mg/mL for Ova-CORT and 0.5–1 mg/mL for BSA-TEST) resulted in an increased background signal in the presence of analytes. Thus, concentrations of 0.5 mg/mL for Ova-CORT and 0.25 mg/mL for BSA-TEST were selected as the optimal concentrations for the immobilized conjugates.

In addition, the effect of the concentrations of immobilized GAMI (in the interception zone) and Protein G (in control line 2) was investigated. A comparison of the signal intensity of control line 2 in the presence and absence of testosterone (200 pg/mL) revealed that the optimal ratio was achieved when Protein G was applied at a concentration of 0.05 mg/mL. To select the optimal GAMI concentration, the color intensity of the interception zone and test line 2 was evaluated (Figure 9c). The selection criterion was the complete absence of a signal on test line 2 when a sample containing the CORT recognition probe was passed through in the absence of both analytes. As shown in Figure 9c, the test strip with 0.5 mg/mL of immobilized GAMI in the interception zone met this criterion.

### 3.5. Analytical Performance of the Duplex LFIA

In the first stage, the analytical characteristics of the proposed duplex LFIA method were evaluated separately for each analyte. As shown in Figure 10a, a decrease in the colorimetric signal at TL1 was observed for cortisol as the analyte concentration increased. At the same time, the intensity of the corresponding interception zone remained virtually unchanged across the concentration range studied. Therefore, for this system, the TL1 intensity was used directly as the analytical signal for quantification, without normalization against the control line. The calculated limit of detection (LOD) and working range for cortisol determination were 0.2 ng/mL and 0.42–5.36 ng/mL, respectively.

For testosterone, a concentration-dependent competitive relationship was observed at TL2, where increasing testosterone levels led to a gradual decrease in TL2 intensity. However, unlike the cortisol detection system, the intensity of the corresponding CL2 also showed a dependence on testosterone concentration; specifically, the colorimetric signal at CL2 increased as the testosterone concentration in the sample rose. Since CL2 was concentration-dependent in this system, it was unsuitable to use CL2 as a standard reference signal. To account for simultaneous changes in the test and control line signals, the TL2/CL2 intensity ratio was used as the analytical parameter for testosterone quantification. This normalization approach resulted in a more pronounced concentration–response relationship and improved assay sensitivity compared to using TL2 intensity alone. In particular, calculating the TL/CL ratio is successfully employed in quantitative competitive LFIA methods, including the analysis of low-molecular-weight compounds and smartphone-based readout systems [53,54,55].

The calibration curve plotting the TL2/CL2 ratio against testosterone concentration, along with digital images of the corresponding test strips, is presented in Figure 10b. The calculated working range for testosterone was 38.4–1259 pg/mL, and the LOD was 13.8 pg/mL.

Thus, although both immunochemical assays were combined into a single duplex LFIA format, different signal processing approaches were applied to them, as the two analytical systems exhibited different signal response patterns. The obtained analytical characteristics demonstrate that both immunochemical systems enable the quantitative determination of cortisol and testosterone across widely differing concentration ranges, corresponding to the conditions required for their detection in saliva.

Subsequently, the simultaneous determination of cortisol and testosterone was performed using a single test strip. Figure 11 shows digital images of the test strips obtained by simultaneously running samples containing both target analytes, along with the corresponding calibration curves. The optimized duplex LFIA provided working ranges of 0.38–5.41 ng/mL for cortisol and 27–845 pg/mL for testosterone, with calculated LODs of 0.18 ng/mL and 10.1 pg/mL, respectively (Table 2). Thus, the LODs for the duplex LFIA are comparable to the analytical performance of the single-analyte assay, demonstrating the successful separation of the two immunochemical reactions on a single membrane.

Next, to verify the reliability of the proposed duplex LFIA, the influence of one analyte on the colorimetric response of the other during simultaneous detection was evaluated. Figure 12a,b demonstrate data obtained for varying concentrations of cortisol/testosterone and constant concentrations of testosterone/cortisol in the samples, respectively. The results indicate the ability of the duplex LFIA method to maintain the response of both analytical systems during simultaneous operation, as well as the high reproducibility of the signal generated by the component present at a constant concentration. Specifically, the standard deviations of the colorimetric response for testosterone and cortisol were 2.8% and 4.6%, respectively.

### 3.6. Specificity

The specificity of the proposed duplex immunochromatographic assay was evaluated by examining the colorimetric response of the immunochemical systems for cortisol and testosterone detection in the presence of potentially interfering steroid hormones, such as estradiol, progesterone, cortisone, and DHEA-sulfate (Figure 13). As shown in Figure 13, the presence of other hormones did not cause a significant reduction in the colorimetric signal, confirming the specificity of the developed assay.

### 3.7. Practical Applicability of Duplex LFIA

Reproducibility was assessed using samples of artificial saliva containing 1 ng/mL of cortisol and 50 pg/mL of testosterone (Figure 14). To evaluate intra-assay reproducibility, each sample was tested using 10 test strips from the same batch under identical conditions. The calculated RSDs for cortisol and testosterone were 4.5% and 5.3%, correspondingly. To evaluate inter-assay reproducibility, samples were tested in a similar manner using test strips from three different batches; the RSDs were 6.7% for cortisol and 6.1% for testosterone. The obtained results demonstrate excellent reproducibility of the duplex LFIA.

The practical applicability of the developed method was evaluated using artificial saliva samples spiked with known concentrations of cortisol and testosterone. Spiked samples were analyzed by duplex LFIA and, in parallel, by a reference ELISA method using a commercial kit for the determination of salivary cortisol and testosterone levels. Table 3 presents the results obtained by the two analytical methods.

The calculated recovery values ranged from 96.4% to 105.7% for cortisol and from 96.9% to 102.8% for testosterone. A comparison of the results obtained using the proposed LFIA method and the reference ELISA showed high agreement for both analytes. Thus, the developed duplex LFIA demonstrated high performance in the analysis of spiked artificial saliva, suggesting its potential for further adaptation toward the study of hormones in saliva.

Table 4 compares the analytical performance of recently developed LFIA-based methods for the determination of cortisol and testosterone. These methods employ various approaches to enhance analytical sensitivity, including signal amplification [29,56], smartphone-based detection [35], photoluminescence [35], and surface-enhanced Raman scattering [33,57,58]. While some of these techniques achieve lower limits of detection, they may necessitate additional signal-processing steps or specialized detection systems. In contrast, the proposed duplex LFIA enables the simultaneous quantitative determination of cortisol and testosterone using a single test strip and a traditional colorimetric readout. Thus, this duplex LFIA offers a practical solution that combines high analytical performance and ease of use while allowing for the simultaneous detection of biomarkers present in a sample at significantly different concentrations.

## 4. Discussion

Modern developments in the field of multiplex immunochromatographic analysis can be categorized into two main strategies for creating test systems: performing the analysis in physically separated flows [30,40,61] or within a single flow on one test strip [62,63,64,65,66] (Table 5).

The design of a multi-LFIA utilizing separate test strips or independent flow channels combined in a single device provides a high degree of physical separation of analytical reactions and the possibility of independent optimization for each analyte. For instance, proposed multiplex LFIA designs [29,40] combine two (or three) independent test strips via a common sample zone, effectively minimizing direct interaction between components. A similar multiplex LFIA design described by Panferov et al. [67] consisted of several separate test strips connected to a common central glass-fiber reservoir for sample application. This device features a radial architecture in which a shared sample application zone serves as the starting point for multiple autonomous analytical channels. However, it should be noted that while these LFIA designs ensure a high degree of separation between immunochemical reactions, they also entail increased device complexity, the use of additional strips or channels, and more sophisticated sample distribution systems.

In contrast, single-membrane multi-LFIAs using a common flow allow the advantages of traditional LFIAs to be retained while simultaneously detecting multiple targets. However, this configuration can affect the analytical performance regarding individual analytes, as immunochemical reactions under flow conditions proceed in a non-equilibrium mode. Bartosh et al. [68,69] demonstrated that altering the arrangement of binding zones in an LFIA designed for the simultaneous detection of four analytes significantly impacts the limits of detection. Optimizing the order of component immobilization on the nitrocellulose membrane resulted in a 2-fold to 27-fold reduction in the limits of detection for individual analytes. In addition to optimizing test line placement, replacing linear detection zones with arrays of spatially separated spots or microarrays [70,71] has been proposed to increase the level of multiplexing within a single flow stream. For instance, Taranova et al. demonstrated a microarray-based test system containing up to 32 test zones (spots) for the simultaneous detection of various analytes [71]. Although this LFIA design allows for a significant increase in the number of test zones on a single membrane, it does not provide selective control over the transfer of recognition reagents. Several studies have proposed placing multiple recognition reagents within a single detection zone and using labels with distinct spectral or other characteristics [66,72]. This architecture enables the detection of a larger number of analytes within a single zone of the test strip, though it imposes additional requirements on signal readout and the equipment used.

The duplex LFIA design proposed in this work represents a different approach within a single membrane architecture, where the introduction of an interception zone between two test lines transforms the standard sequential multiplex LFIA within one flow into a functionally separated system. Consequently, compared to previously described multiplex LFIA tests, the proposed design retains the compactness and visual simplicity of single-nitrocellulose-membrane systems and the traditional gold-nanoparticle detection method while ensuring spatial selectivity to prevent cross-interference between recognition probes.

**Table 5:** Comparison of multiplex LFIA designs.

Main Principle	Main Advantage	Principle Limitation	Ref.
**Multiple LFIA analysis in physically separated flow**			
Several independent test strips in a single LFIA T1-C1/T2-C2/T3-C3	Strong isolation of assays; Each assay can be independently optimized; High sensitivity; Simultaneous analysis from one sample injection.	Larger device; Multiple flow paths; Potentially greater sample requirement; Requires several channels.	[30,40,61,67]
**Multiple test lines on one strip**			
Multiple-line strip T1–T2–T3–C	Simplest and most established multiplex format; Compact and inexpensive.	Upstream/downstream interactions; Performance depends on line position and order; Reporter conjugates coexist throughout the flow path.	[62,63,68,69,73]
Identification using multiple spectrally distinct labels	Very compact; Potentially high multiplexing capacity.	Requires distinct signals; Potentially more complex detection equipment; More sophisticated reporters and readers.	[64,65,66,72,74]
Microarrays on the one strip	High multiplexing capacity.	Test zones remain exposed to the common flow; Flow distribution and reaction kinetics may affect performance.	[70,71]
**Multiple-line strip with reporter interception zone**			
TL1– interception zone–TL2–CL2 on one membrane	Single working membrane; Conventional colorimetric readout; No spectrally distinct labels; Prevention of reporter cross-interference.	Currently demonstrated for two analytes; Additional interception zones would require further validation and optimization.	**This ** **work**

## 5. Conclusions

In this study, we designed a duplex lateral flow immunoassay featuring the interception zone positioned between the reaction zones for cortisol and testosterone. This zone selectively removes the residual CORT recognition probe while allowing the TEST recognition probe to reach the subsequent reaction zone, thereby minimizing cross-interference between the components of the simultaneous immunoreactions occurring on a single membrane.

Under optimized conditions, the duplex lateral flow immunoassay enabled the quantitative determination of cortisol and testosterone in artificial saliva across concentration ranges relevant for assessing endocrine status. For simultaneous determination, the working ranges were 0.38–5.41 ng/mL for cortisol and 27–845 pg/mL for testosterone, with limits of detection of 0.18 ng/mL and 10.1 pg/mL, respectively.

The developed test system also demonstrated specificity in the presence of potentially interfering steroid hormones, including estradiol, progesterone, cortisone, prednisone, and DHEA-sulfate. Analysis of spiked artificial saliva yielded recovery rates of 96.4–105.7% for cortisol and 96.9–102.8% for testosterone, alongside good agreement with results obtained via the reference ELISA method.

In summary, these findings indicate that the spatial organization of immunochemical reactions, combined with the capture of the interfering recognition probe, offers a practical approach to developing multiplex lateral flow immunoassays, particularly when analytes exhibit vastly different concentration ranges and the use of a single colorimetric label is preferred. The proposed design retains the simplicity of a single-membrane gold nanoparticle-based lateral flow immunoassay while simultaneously reducing cross-interference between recognition probes and eliminating the need for spectrally distinct labels.

## Figures and Tables

**Figure 1:**
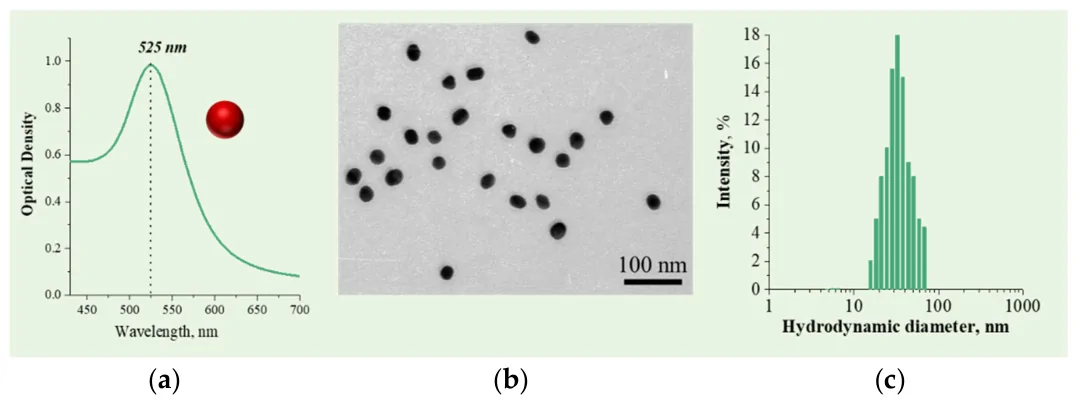
Absorption spectrum (**a**), TEM image (**b**) and histogram of nanoparticle size distribution (**c**) registered for AuNPs.

**Figure 2:**
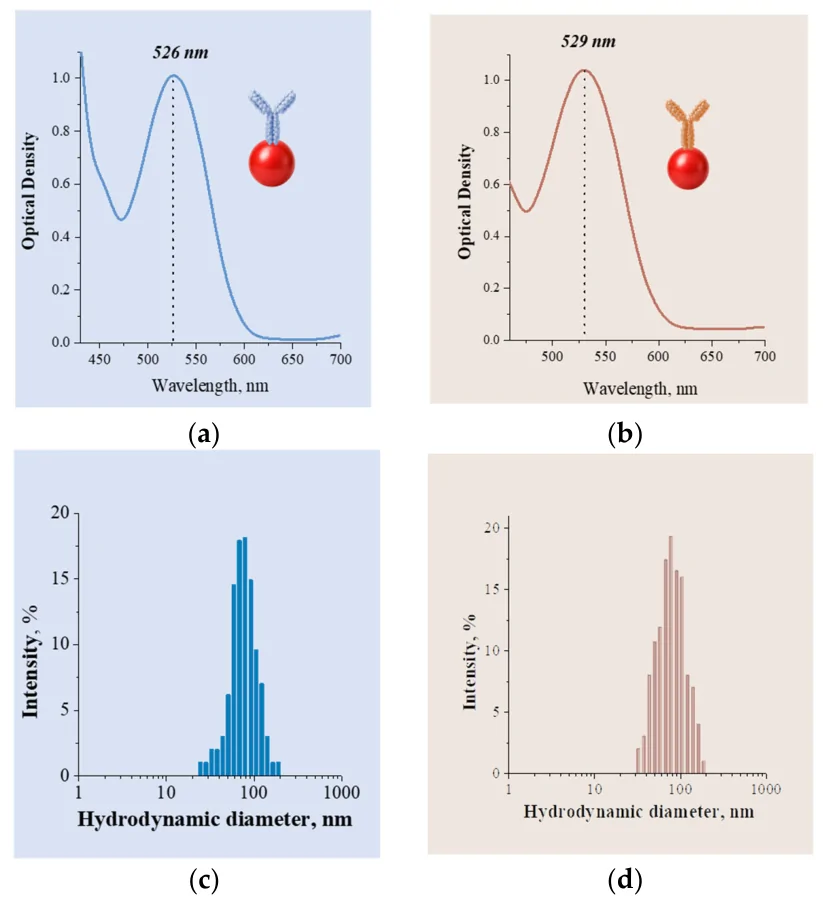
Absorption spectra (**a**,**b**) and histograms of nanoparticle size (**c**,**d**) distribution registered by DLS registered for CORT recognition probe (**a**,**c**) and TEST recognition probe (**b**,**d**).

**Figure 3:**
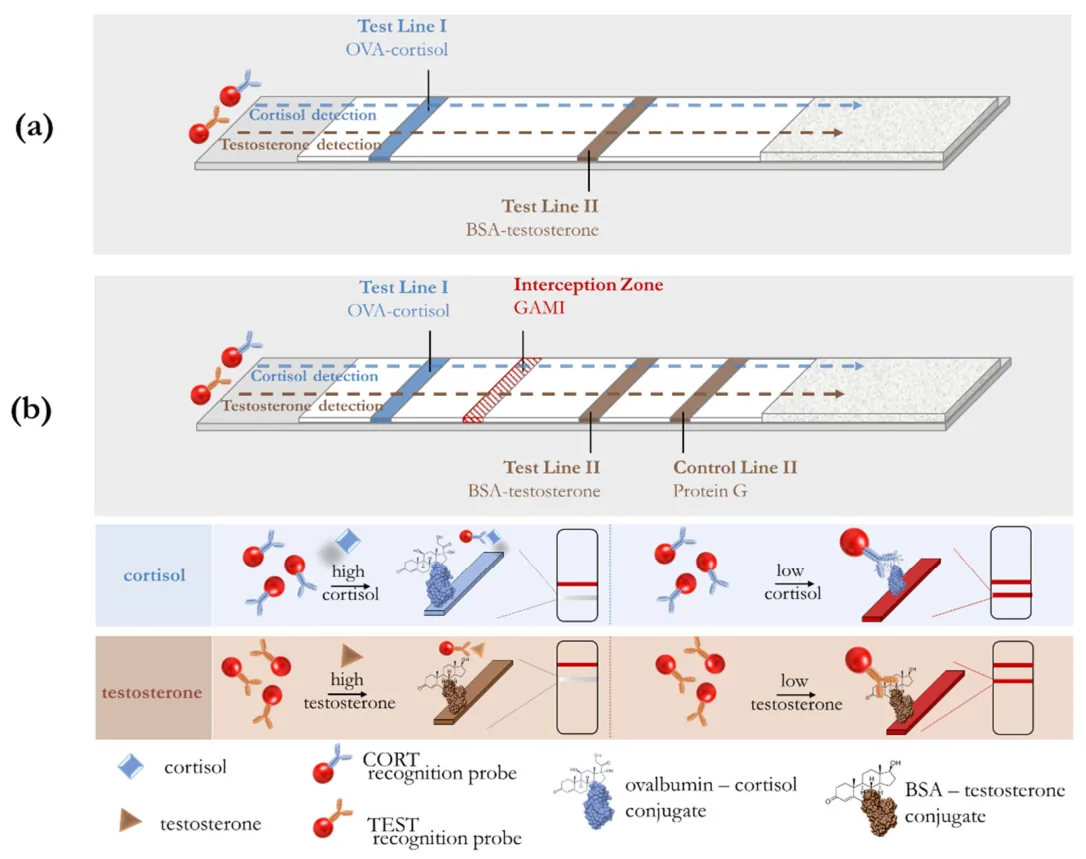
(**a**) Traditional competitive LFIA using a single flow on a single test strip for the simultaneous detection of cortisol and testosterone. (**b**) Scheme of duplex lateral flow immunoassay for simultaneous colorimetric determination of cortisol and testosterone. The LFIA test strip has four binding zones (from bottom to top): (1) test line I containing CORT–ovalbumin conjugate for cortisol detection; (2) an interception zone containing GAMI for capturing an unbound CORT recognition probe; (3) test line II containing TEST-BSA conjugate for testosterone detection; and (4) control line II containing Protein G for capturing an unbound TEST recognition probe.

**Figure 4:**
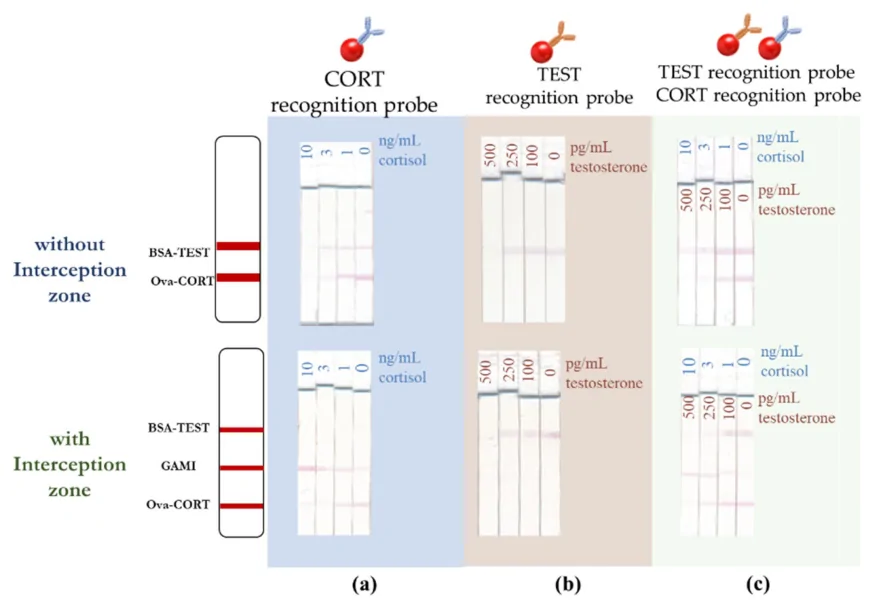
Testing of the interception zone for spatial separation of immunochemical reactions under different conditions: (**a**) passing CORT recognition probe in the presence and absence of 10 ng/mL cortisol; (**b**) passing TEST recognition probe in the presence and absence of 500 ng/mL testosterone; (**c**) passing CORT recognition probe and TEST recognition probe in the presence and absence of 500 ng/mL testosterone and 30 ng/mL cortisol.

**Figure 5:**
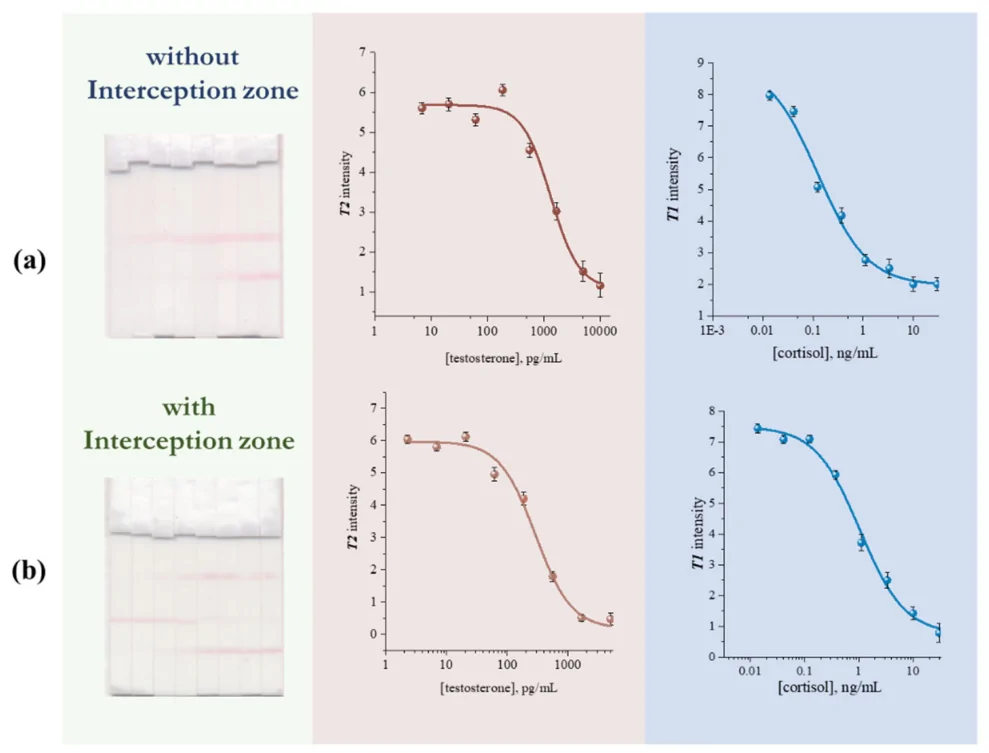
Calibration curves for cortisol and testosterone detection using duplex LFIA without (**a**) and with (**b**) interception zone.

**Figure 6:**
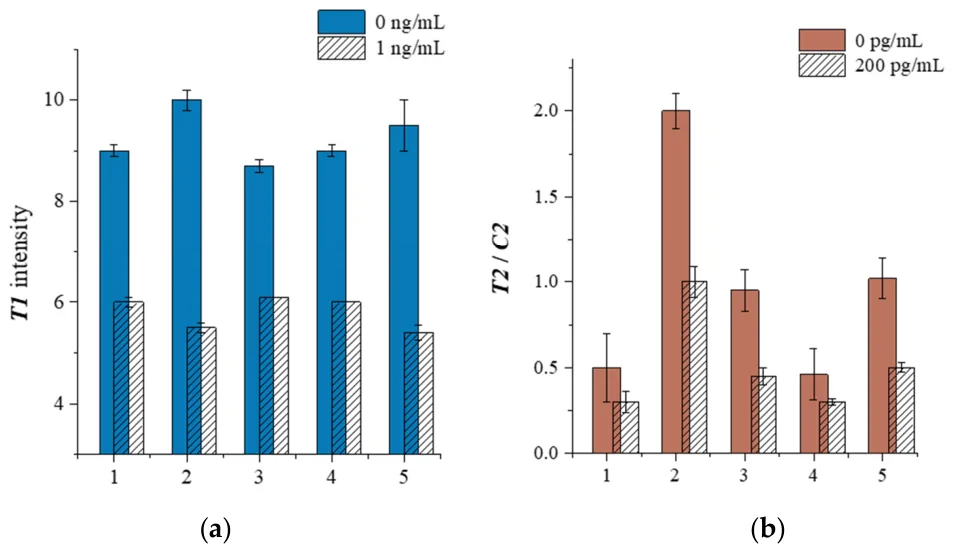
Colorimetric responses obtained after performing duplex LFIA for cortisol (**a**) and testosterone (**b**) using various saliva dilution buffers: ***1***—Tris-HCl (20 mM, pH 8.7), containing 150 mM NaCl and 0.5% Triton X-100; ***2***—PBS (10 mM, pH 7.4), containing 150 mM NaCl and 0.5% Tween-20; ***3***—HEPES (10 mM, pH 8.1), containing 150 mM NaCl and 0.5% Tween-20; ***4***—Tris-HCl (20 mM, pH 8.7), containing 150 mM NaCl and 0.5% Tween-20; and ***5***—PBS (10 mM, pH 7.4) and Tris-HCl (20 mM, pH 8.7), containing 150 mM NaCl, 0.5% Tween-20 and 0.5% Triton X-100. The concentrations of the positive samples were 1 ng/mL for cortisol and 200 pg/mL for testosterone.

**Figure 7:**
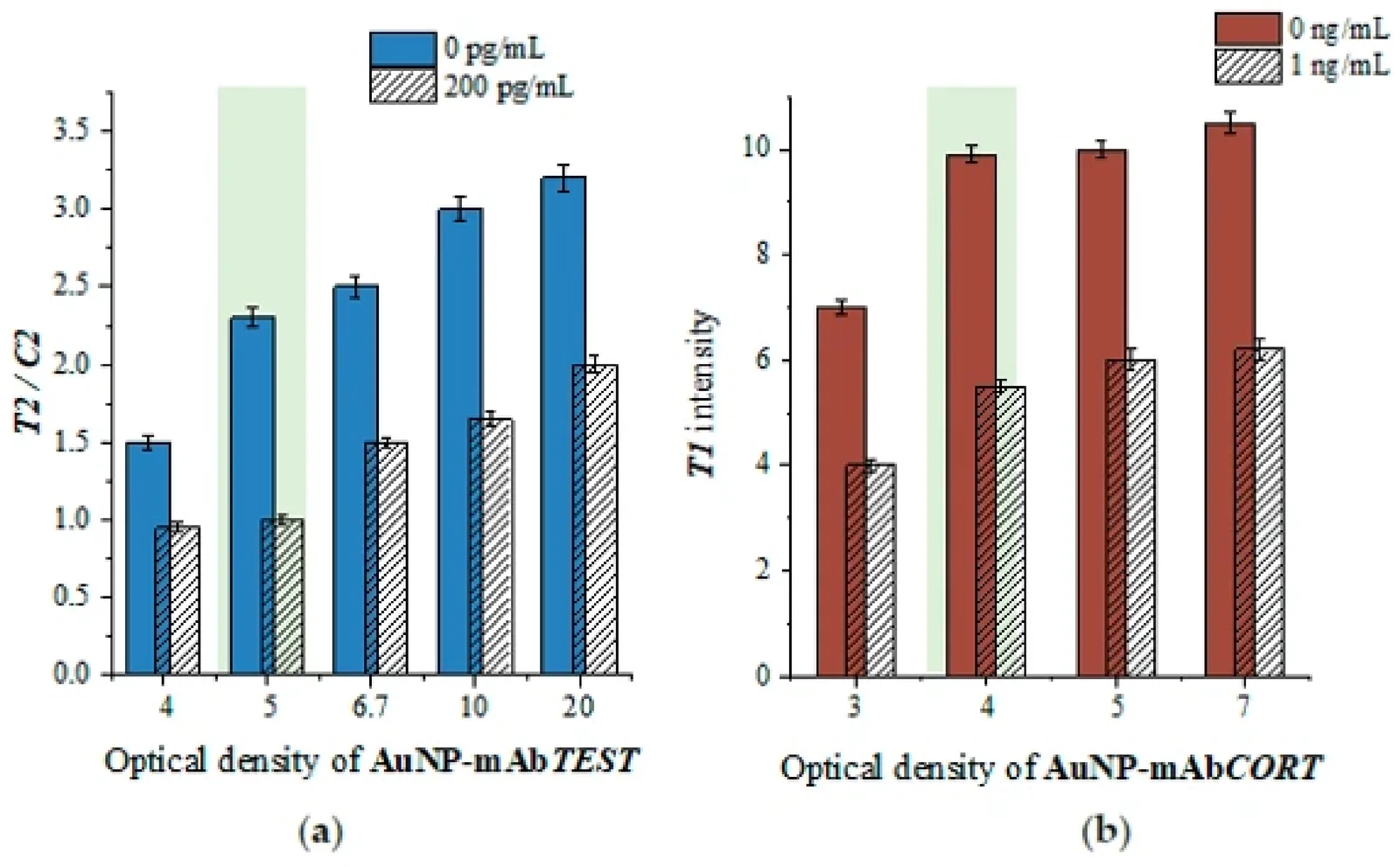
Colorimetric responses obtained using a duplex LFIA with varying optical densities of the TEST recognition probe (**a**) and CORT recognition probe (**b**). The concentrations of the positive samples were 1 ng/mL for cortisol and 200 pg/mL for testosterone.

**Figure 8:**
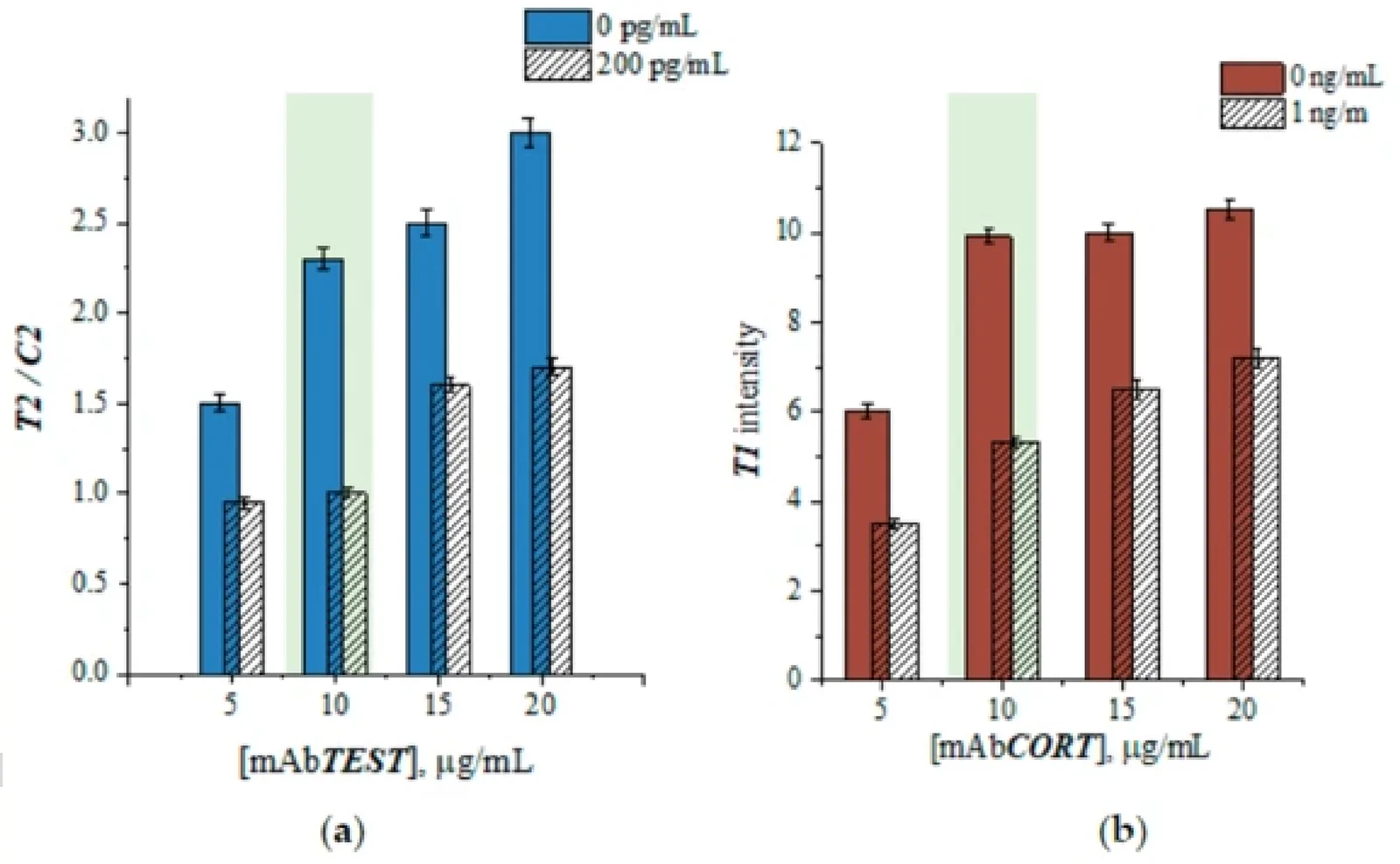
Colorimetric responses obtained after performing duplex LFIA with varying antibody concentrations in the TEST recognition probe (**a**) and CORT recognition probe (**b**). The concentrations of the positive samples were 1 ng/mL for cortisol and 200 pg/mL for testosterone.

**Figure 9:**
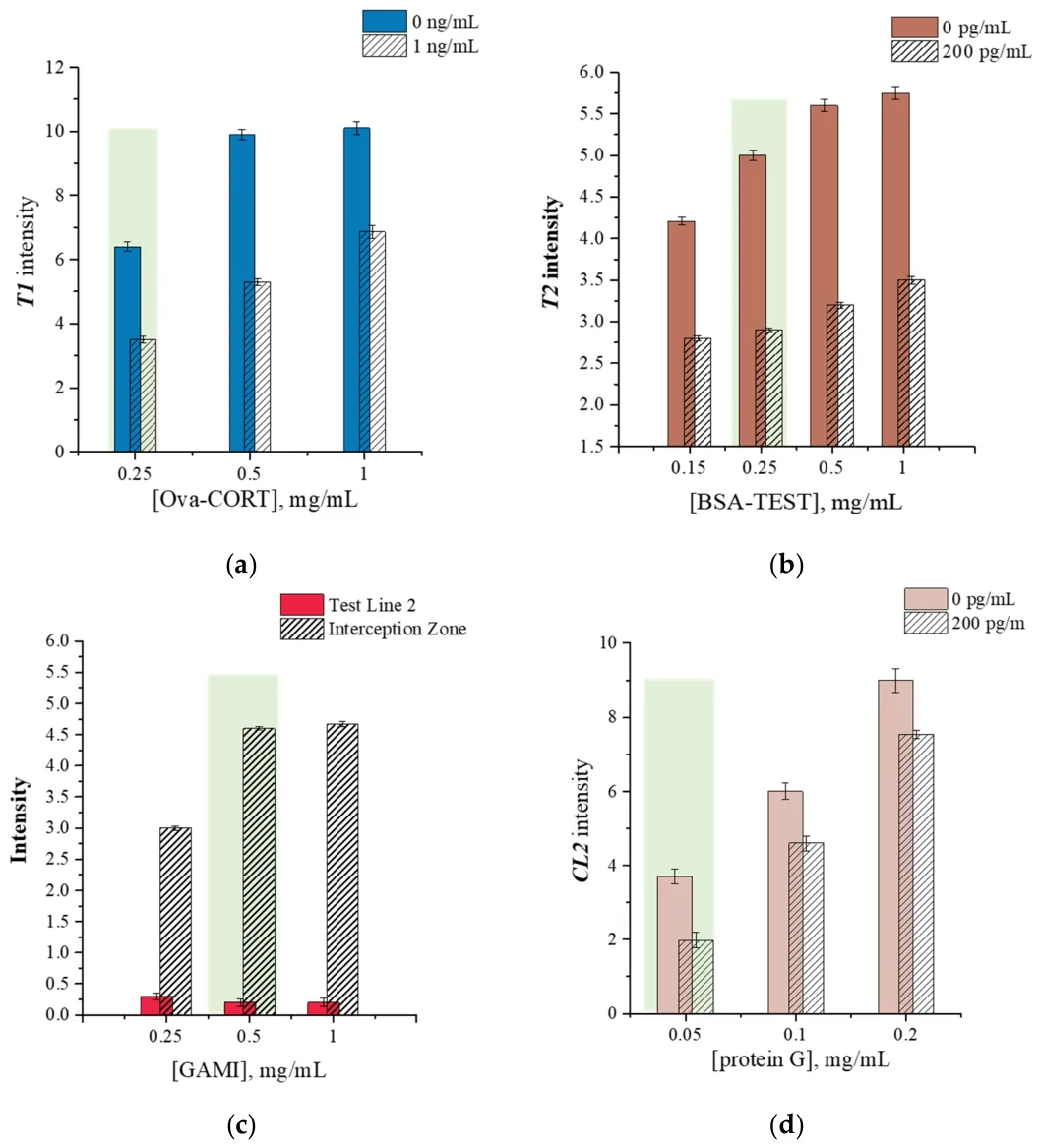
Optimization of the concentration of immobilized components in various analytical zones on the nitrocellulose membrane. (**a**) Intensity of test line 1 obtained in the presence and absence of 1 ng/mL cortisol for various concentrations of the ovalbumin–cortisol conjugate. (**b**) Intensity of test line 2 obtained in the presence and absence of 200 pg/mL testosterone for various concentrations of the BSA–testosterone conjugate. (**c**) Color intensity of the interception zone obtained after passing the CORT recognition probe—in the absence of both analytes—over various concentrations of GAMI. (**d**) Intensity of control line 2 obtained in the presence and absence of 200 pg/mL testosterone for various concentrations of Protein G.

**Figure 10:**
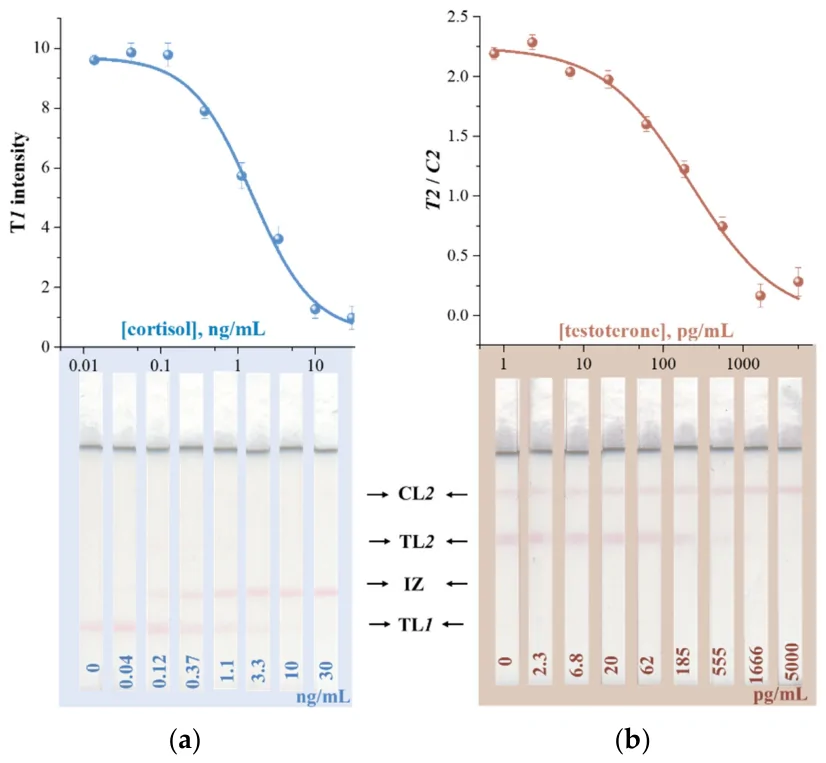
(**a**) Calibration curve for cortisol detection obtained by duplex LFIA. Inserts: digital images of LFIA test strips obtained for 0, 0.04, 0.12, 0.37, 1.1, 3.3, 10 and 30 ng/mL of cortisol. (**b**) Calibration curve for testosterone detection obtained by duplex LFIA. Inserts: digital images of LFIA test strips obtained for 0, 2.3, 6.8, 20, 62, 185, 555, 1666 and 5000 ng/mL of testosterone. The error bars shown in the graph were estimated based on three repeats of analysis.

**Figure 11:**
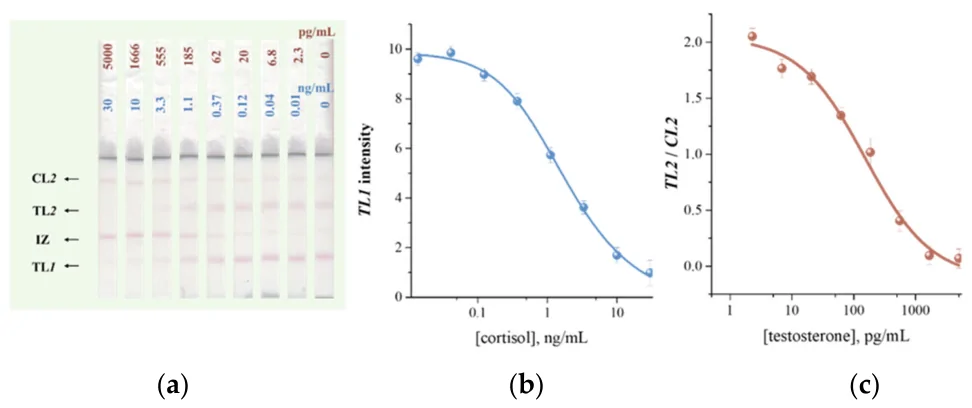
(**a**) Digital images of LFIA test strips obtained for 0, 2.3, 6.8, 20, 62, 185, 555, 1666 and 5000 ng/mL of testosterone and 0, 0.01, 0.04, 0.12, 0.37, 1.1, 3.3, 10 and 30 ng/mL of cortisol. (**b**) Calibration curve for cortisol detection obtained by duplex LFIA. (**c**) Calibration curve for testosterone detection obtained by duplex LFIA. The error bars shown in the graph were estimated based on three repeats of analysis.

**Figure 12:**
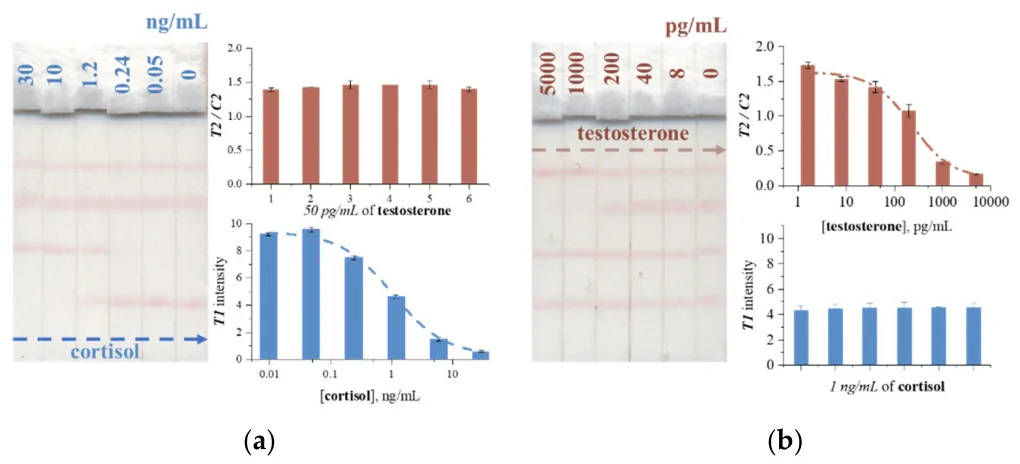
Testing of the developed duplex LFIA under the following conditions: (**a**) varying cortisol concentration in the range of 0–30 ng/mL at a constant testosterone concentration of 50 pg/mL; (**b**) varying testosterone concentration in the range of 0–5000 pg/mL at a constant cortisol concentration of 1 ng/mL.

**Figure 13:**
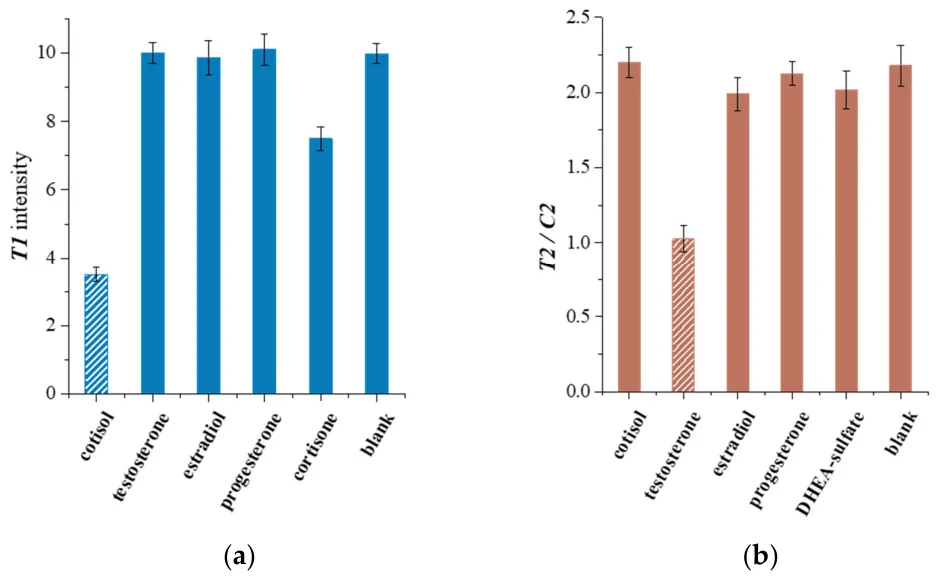
Specificity testing of the duplex LFIA in the presence of other interfering hormones: (**a**) the colorimetric signal measured in the TL1 after application of different hormones; (**b**) the colorimetric signal measures in the testosterone detection zone after application of different hormones.The concentration of cortisol is 3 ng/mL, concentration of testosterone is 100 pg/mL, and concentration of other hormones is 10 ng/mL.

**Figure 14:**
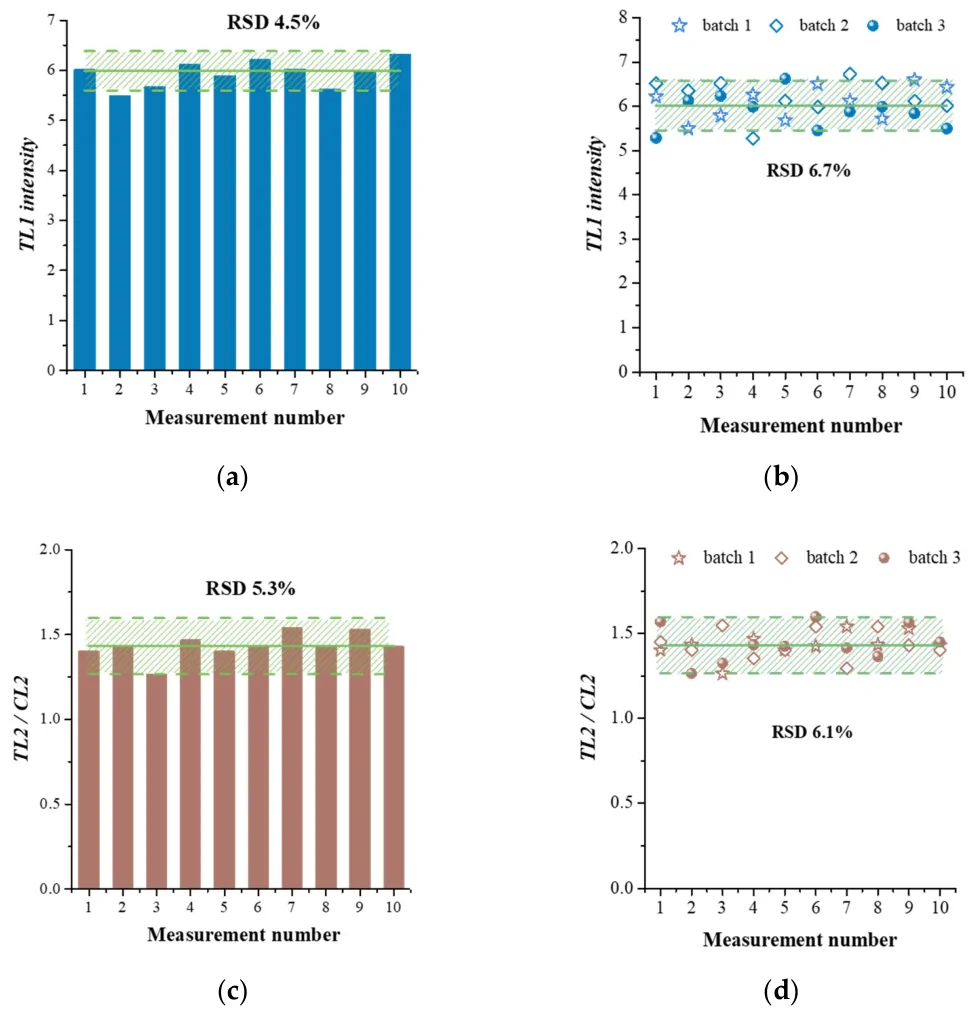
Intra- (**a**,**c**) and inter-batch (**b**,**d**) reproducibility of the colorimetric signal obtained for 1 ng/mL cortisol (**a**,**b**) and 50 pg/mL testosterone (**c**,**d**). The number of batches is 3.

**Table 1:** Comparison of the analytical performances of cortisol and testosterone detection by duplex LFIA with and without interception zone.

Analyte	Parameters	Without IZ	With IZ
cortisol	LOD, ng/mL	0.01	0.16
	Working range, ng/mL	0.03–0.6	0.32–3.9
	EC50, ng/mL	0.1	1.0
	CV, %	4.0	3.9
	CI *, high/low **, %	4.5	4.4
	CI, low/high ***, %	3.9	3.7
testosterone	LOD, ng/mL	0.4	0.06
	Working range, ng/mL	0.6–2.9	0.11–0.75
	EC50, ng/mL	1.3	0.3
	CV, %	3.2	2.0
	CI, high/low, %	13.8	3.4
	CI, low/high, %	4.9	4.6

* Cross-interference (CI) was determined by comparing the signal obtained when testing the analyte individually with the signal obtained from a mixture of two analytes. ** ***high/low—***c(CORT) 5 ng/mL c(TEST) 50 pg/mL. *** ***low/high—***c(CORT) 1 ng/mL c(TEST) 500 pg/mL.

**Table 2:** Comparison of the analytical performance of duplex LFIA for cortisol and testosterone upon their individual and simultaneous determination.

Analyte		Detection Limit	Detection Range
Individual determination	cortisol	0.2 ng/mL	0.42–5.36 ng/mL
	testosterone	9.5 pg/mL	25.4–795 pg/mL
Simultaneous determination	cortisol	0.18 ng/mL	0.38–5.41 ng/mL
	testosterone	10.1 pg/mL	27–845 pg/mL

**Table 3:** Testing of artificial saliva samples spiked with cortisol and testosterone using the developed duplex LFIA assay and a reference ELISA.

**cortisol**	**Concentration, ng/mL**	**Detected by LFIA, ng/mL**	**Recovery, %**	**Detected by ELISA, ng/mL**	**Recovery, %**
	0.35	0.37 ± 0.12	105.7	0.36 ± 0.09	102.8
	2.25	2.17 ± 0.20	96.4	2.23 ± 0.12	99.1
**testosterone**	**Concentration, pg/mL**	**Detected by LFIA, pg/mL**	**Recovery, %**	**Detected by ELISA, pg/mL**	**Recovery, %**
	65	63.0 ± 1.6	96.9	66.2 ± 0.8	101.8
	250	257.0 ± 2.4	102.8	248.2 ± 2.1	99.2

**Table 4:** A comparison of recent developments based on LFIA for the determination of cortisol and testosterone.

Assay	Detection Limit, ng/mL	Range, ng/mL	Matrix	Dilution Factor	Ref.
**Cortisol**					
automatically signal-enhanced LFIA	0.038	0.01–10	saliva	10	[29]
paper-based trap LFIA	0.0091	0.01–1000 ng/mL	saliva, serum, and urine samples	10	[56]
immune-chromatographic strip	1	1–100 ng/mL	human saliva	undiluted	[59]
smartphone-based photoluminescence LF IA	0.139 ng/mL	0.14–200 ng/mL	human saliva	n/a	[35]
microchannel lateral flow assay on lab-on-a-chip	1.8	0.93–30.0	spiked artificial saliva	2	[60]
lateral flow immunoplatform integrated with surface-enhanced Raman scattering	0.014 × 10^−3^	0.0001–100	human saliva	10	[57]
Dual-Competitive Turn-On SERS-LFIA	0.003	0.01–100	human saliva	10	[33]
**Testosterone**					
Au@Au SERS nanolabel-based LFIA	2.27 × 10^−6^	10 × 10^−6^–10 × 10^3^	human serum	undiluted	[58]
**Cortisol and testosterone**					
duplex LFIA	0.18 for cortisol 10.1 × 10^−3^ for testosterone	0.38–5.41 ng/mL for cortisol 27 × 10^−3^–845 × 10^−3^ for testosterone	spiked artificial saliva	2	This study

## Data Availability

The original contributions presented in this study are included in the article. Further inquiries can be directed to the corresponding author.
